# Dispersion Interactions between Molecules in and out of Equilibrium Geometry: Visualization and Analysis

**DOI:** 10.1021/acs.jpca.2c00004

**Published:** 2022-02-15

**Authors:** Piotr H. Kowalski, Agnieszka Krzemińska, Katarzyna Pernal, Ewa Pastorczak

**Affiliations:** Institute of Physics, Lodz University of Technology, ul. Wolczanska 217/221, 93-005 Lodz, Poland

## Abstract

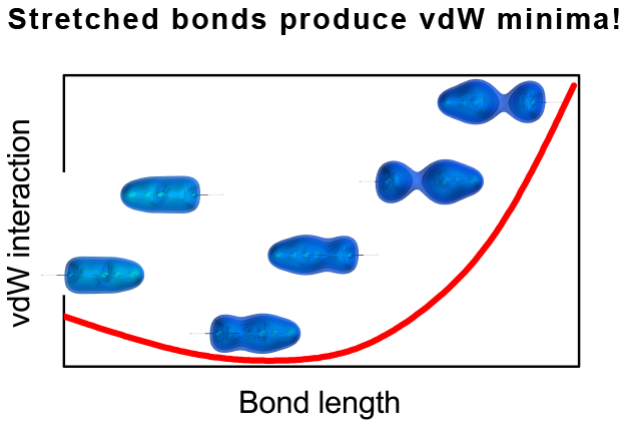

The London dispersion interactions between systems undergoing bond breaking, twisting, or compression are not well studied due to the scarcity and the high computational cost of methods being able to describe both the dynamic correlation and the multireference character of the system. Recently developed methods based on the Generalized Valence Bond wave function, such as EERPA-GVB and SAPT(GVB) (SAPT = symmetry-adapted perturbation theory), allow one to accurately compute and analyze noncovalent interactions between multireference systems. Here, we augment this analysis by introducing a local indicator for dispersion interactions inspired by Mata and Wuttke’s Dispersion Interaction Density [J. Comput. Chem.2017, 38, 15−2327761924] applied on top of an EERPA-GVB computation. Using a few model systems, we show what insights into the nature and evolution of the dispersion interaction during bond breaking and twisting such an approach is able to offer. The new indicator can be used at a minimal cost additional to an EERPA-GVB computation and can be complemented by an energy decomposition employing the SAPT(GVB) method. We explain the physics behind the initial increase, followed by a decrease in the interaction of linear molecules upon bond stretching. Namely, the elongation of covalent bonds leads to the enhancement of attractive dispersion interactions. For even larger bond lengths, this effect is canceled by the increase of the repulsive exchange forces resulting in a suppression of the interaction and finally leading to repulsion between monomers.

## Introduction

The London dispersion, the most elusive of the noncovalent interactions, is known to play a key role in the formation of molecules,^1^ reaction barrier heights,^2^ and catalytic processes^3^ as well as in the design of one- and two-dimensional van der Waals heterostructures.^4^ While modern computational methods are able to capture the London dispersion interaction accurately, separating dispersion from other effects is still challenging: the symmetry-adapted perturbation theory (SAPT)^5,6^ methods are able to rigorously isolate the dispersion contribution to the total energy of the interaction between systems, but only recently their capabilities are being extended to intramolecular^7,8^ and multireference cases.^9−11^

Another group of tools for analyzing the noncovalent interactions are the visualization indicators, such as Non-Covalent Interactions Index (NCI),^12^ QTAIM-based tools,^13^ or DORI,^14^ where usually the relative strengths of interactions are associated with molecular fragments. Such indicators are useful thanks to their intuitive nature and a relatively broad range of applicability: they can be employed both in the inter- and intramolecular cases, in large systems.

There are a couple of ways to isolate and map specifically the dispersion interaction in physical space, namely, the London dispersion potential (LDP) maps of Pollice and Chen^15^ and the Dispersion Interaction Density (DID), based on the second-order spin-component scaled^16^ local Møller–Plesset method (LMP2), proposed by Mata and Wuttke.^17^ The latter method utilizes the localized nature of correlation contributions in the LMP2 method and identifies the parts of the correlation energy responsible for the dispersion interaction. This approach is consistent with the SAPT definition of dispersion and is computationally efficient, so it has been very helpful in understanding the interaction inside large molecules.^18^ However, as single-reference approaches, neither LDP nor DID allow for studying systems, where covalent bonds are significantly stretched or compressed. Such systems include interacting materials under pressure, stretched nanowires and nanoribbons, and molecules approaching chemical reactions; therefore, a tool able to get insight into those phenomena could prove very useful.

To be able to adapt the DID approach for those challenging cases, one needs a computationally efficient multireference approach in which the dispersion contribution can be isolated from the correlation energy, and it can be assigned to physical domains consisting of molecules or molecular fragments. One approach fulfilling those requirements is a variant of the generalized valence bond method (namely, the strongly orthogonal perfect-pairing Generalized Valence Bond, in the rest of the paper denoted as GVB) amended by a correlation correction based on the Extended Random Phase Approximation (ERPA).^19−21^

The purpose of this work is to introduce a quantity analogous to Mata and Wuttke’s DID based on the ERPA-GVB method and show the robustness of the new indicator on a few model systems including dimers whose components are out-of-equilibrium molecules. In the following section, we will show how to extract the dispersion component from the correlation energy in ERPA (and Embedding ERPA) approach. In the Results and Discussion section we will present the new indicator being applied to examples of interactions in water, ethylene, acetylene, and diacetylene dimer in different geometries. This picture will be complemented by a symmetry-adapted perturbation theory computation decomposing the interactions into physically meaningful terms. Finally, we will discuss how the obtained results fit in with the current state of knowledge on noncovalent interactions and with our previous observations.

## The Dispersion Energy Indicator

The GVB ansatz reads^22−24^
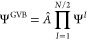
1where *Â* denotes the antisymmetrization operator, *N* is the (even) number of electrons, and Ψ^*I*^ are GVB geminals, that is, antisymmetric singlet-coupled two-electron wave functions
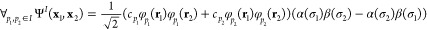
2strongly orthogonal to each other. A natural orbital representation is employed throughout this work; thus, a coefficient *c*_*p*_ is directly related to a corresponding spin-orbital occupation number *n*_*p*_ as follows.
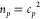
3

The index *p* enumerates the orbitals, and, for orbitals comprising the *I*th geminal, it corresponds to *p*_1_ and *p*_2_ indices in eq 2.

The GVB electronic energy reads^21,24^
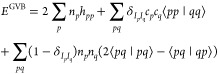
4with {*h*_*pp*_ = ⟨φ_*p*_|*t̂* + υ̂_ext_|φ_*p*_⟩} being one-electron integrals in natural-orbitals representation, and symbols *I*_*p*_ in eq 4 indicate a geminal to which an orbital φ_*p*_ belongs. The GVB energy lacks the intergeminal correlation energy. This shortcoming can be overcome, for example, by adding a correlation correction^20,21,25^ derived by employing the ERPA equations.^19,26,27^
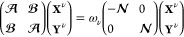
5

The eigenvectors [**X**^ν^, **Y**^ν^] approximate the reduced transition density matrices, which allows one to write the spin-summed ERPA correlation energy expression as^20,21,28^
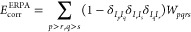
6
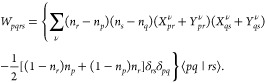
7

The term (1 – δ_*I*_*p*_*I*_*q*__δ_*I*_*r*_*I*_*s*__δ_*I*_*q*_*I*_*r*__) in eq 6 vanishes when all orbitals *p*, *q*, *r*, *s* belong to the same geminal. Thus, by construction there is no double counting of correlation if the correlation energy *E*_corr_^ERPA^ is added to the GVB energy *E*^GVB^.

Since electrons belonging to different geminals are uncorrelated, then if the ERPA-GVB approach is employed for weakly interacting systems, the total contribution from the dispersion interaction is comprised solely within the ERPA correction. When it comes to a computation of interaction energies of dimers, the ERPA description of the correlation energy for a dimer is poorer than that of a monomer, since some of the orbitals unoccupied in the monomer computation become fractionally occupied in a dimer computation. This leads to eliminating many components in the sum in eq 6 and, consequently, to a less accurate description of the dimer’s correlation energy. This deficiency of ERPA can be alleviated by treating each of the monomers comprising the dimer as a subsystem embedded in the field of the other monomer. This “Embedding ERPA” (EERPA) approach allows for a richer and more balanced description of the electron correlation.^25,29^ As shown in ref (30), the EERPA approach can be extended to the treatment of electrons belonging (in the sense of the localized GVB orbitals) to distinct fragments of molecules as subsystems embedded in the field of the remaining fragments. The fragments can be chosen arbitrarily; for example, a single geminal can be treated as a subsystem.^30^ It is worth reiterating that partitioning of GVB orbitals into unequivocal fragments of molecular systems is feasible due to the localized nature of both strongly and weakly occupied GVB orbitals. In this work, the variant of EERPA assuming a partitioning of the GVB orbitals into two fragments *A* and *B* associated with distinct monomers is used.

The EERPA correlation energy of a dimer *AB* can be partitioned into one-body terms pertaining to intramonomer correlation and the remaining two-body term.^25,29^
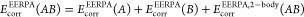
8

In the context of dispersion interaction we are interested in terms *W*_*pqrs*_ (see eq 6) in a two-body *E*_corr_^EERPA, 2–body^(*AB*) term, which are least-vanishing in the limit of intermonomer distance *R*_*AB*_ approaching infinity. By repeating the analysis from ref (27), it is straightforward to show that the energy
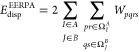
9where *I* and *J* indicate geminals, the set Ω_*I*_^*A*^ comprises occupied orbitals from a geminal *I*, localized on the monomer *A*, virtual orbitals and weakly occupied  orbitals localized on *B*
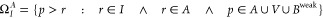
10
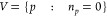
11
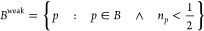
12(analogously for Ω_*J*_^*B*^), tends asymptotically to the dispersion energy expression reading
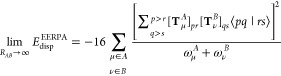
13

The latter is given in terms of monomer properties: transition energies ω_μ_^*A*/*B*^ and transition density matrices **T**_μ_^*A*/*B*^ corresponding to solutions of the ERPA equations, cf. eq 5, for isolated monomers, in particular
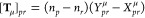
14

Notice that dispersion energy as given in eq 9 is determined by the response of a dimer; that is, the summation with respect to ν in eq 7 accounts for all excited states of a dimer predicted by the ERPA equations. In the dissociation limit, each state ν becomes a composition of states of monomers, cf. eq 13.

Let us introduce the intergeminal correlation terms as
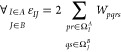
15where geminals *I* and *J* are assumed to be localized on distinct fragments. A sum of such terms yields EERPA dispersion-like correlation energy, cf. eq 9
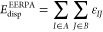
16and each ε_*IJ*_ term has the interpretation of the dispersion interaction between geminals *I* and *J*.

Having identified intergeminal components of the correlation energy ε_*IJ*_ giving rise to dispersion energy, we follow Wuttke and Mata^17^ and define a local descriptor of dispersion on a fragment *A* interacting with a fragment *B* as
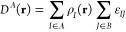
17and for *B* interacting with *A*
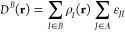
18or
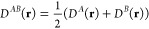
19where a geminal density ρ_*I*_ reads
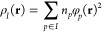
20

Since each geminal is normalized to one, that is
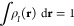
21it follows trivially that integration of *D*^*A*^(**r**) or, equivalently, *D*^*B*^(**r**) leads to obtaining the dispersion energy in the limit when monomers do not overlap, namely
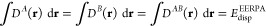
22and the limiting expression is given by eq 13. This property shows that quantities *D*^*A*/*B*/*AB*^(**r**) can be viewed as the dispersion interaction energy densities.

Finally, it is worthwhile noticing that, by exploiting the localized nature of geminals, partitioning the set of geminals into interacting fragments can be applied for a single molecule. In this case, *A* and *B* would be parts of the same molecule; the descriptors *D*^*X*^(**r**) and the energy *E*_disp_^EERPA^ would correspond to the intramolecule dispersion energy.

## Results and Discussion

In principle, either of the EERPA-GVB-based dispersion descriptors *D*^*A*^, *D*^*B*^, or *D*^*AB*^ could be employed to identify the sources of the dispersion interaction. However, only the latter treats both monomers on equal footing, and therefore it will be used throughout the paper. To demonstrate the usefulness of the indicator *D*^*AB*^ we will present its performance for a number of weakly interacting dimers in and out of their equilibrium geometries.

In order to provide more quantitative insight into the character of molecular interactions in systems with stretched covalent bonds, we will use the recently proposed multiconfigurational symmetry adapted perturbation theory SAPT(MC).^9−11^ This method predicts molecular interaction energy up to the second-order terms in the interaction operator
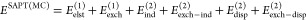
23

In SAPT(MC) the exchange components employ the *S*^2^ approximation, and the density–density response function entering the second-order terms is described at the ERPA level. Only one- and two-electron reduced density matrices of monomers, obtained from multiconfigurational wave functions describing monomers, are needed in SAPT(MC). While other SAPT methods are likely to fail when bonds are strongly elongated in monomers, SAPT(MC) stays reliable.^9^ This unique feature of the method makes it a proper tool to study systems discussed in this work. A consequence of employing GVB reduced density matrices in SAPT(MC), denoted as SAPT(GVB), is the consistency of the dispersion energy from EERPA, see eqs 9 and (13), integrated dispersion descriptors, see eq 22, and *E*_disp_^(2)^, that is
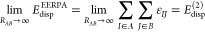
24

The equilibrium geometries of studied complexes were taken from the Nonbonded Interactions database published by Truhlar et al.,^31^ and the calculations were performed in the aug-cc-pVDZ basis set,^32^ except for the diacetylene dimer, for which the equilibrium geometry was taken from ref (33), and the basis set used was 6-311++g(d,p) employed in the same work. For acetylene and ethylene dimer the intermonomer distances were kept fixed, while for diacetylene the intermonomer distances were changed, but the molecular structures were kept frozen. The computations of the dispersion indicator, EERPA-GVB, and SAPT(GVB) energies were performed using the in-house developed GammCor code^34^ interfaced with the DALTON software package.^35^ The reference CC3 computations were also performed using the DALTON package. The visualizations were realized using a code developed by us in Python,^36^ with the help of a Mayavi^37^ libraries package. The developed code is available at ref (38).

**Figure 1:**
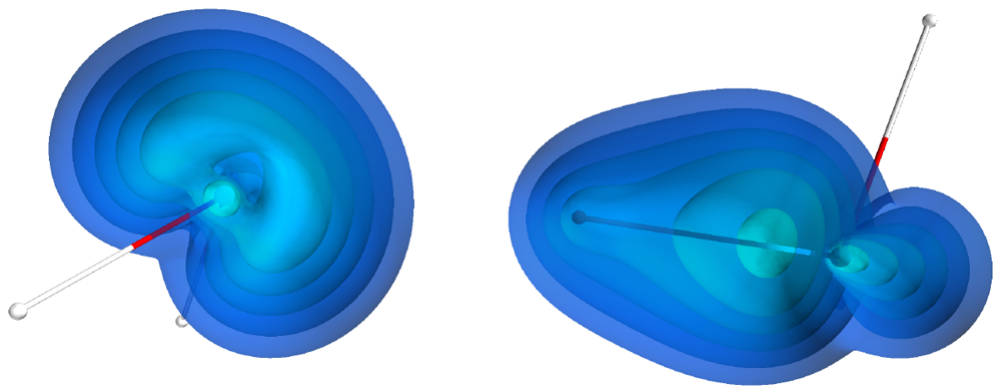
Visualization of the dispersion interaction density *D*^*AB*^ for water dimer. The six contours of isosurfaces of *D*^*AB*^(**r**) densities were generated to encompass 50%, 40%, 30%, 20%, 10%, and 1% of the integrated *D*^*AB*^(**r**), respectively.

As the first example of visualization of the dispersion, we chose a water dimer (see Figure 1). This system is considered a textbook example for hydrogen bonds,^39^ and therefore the dominant interaction between the water molecules is electrostatic; however, dispersion accounts for ∼30% of the binding.^40^ The dispersion indicator correctly identifies the main interacting areas as the OH bond in one molecule and the electron lone pair on the other. The picture provided by the indicator is consistent with the one produced by such tools as NCI^41^ and DORI^14^ and very similar to the one presented by VSEPR, an analysis tool based on Lewis pairs,^42^ which underlines the electron-pair-based character of our indicator.

The real strength of the indicator, however, lies in the description of dispersion-bound systems containing molecules out of their equilibrium geometry.

**Figure 2:**
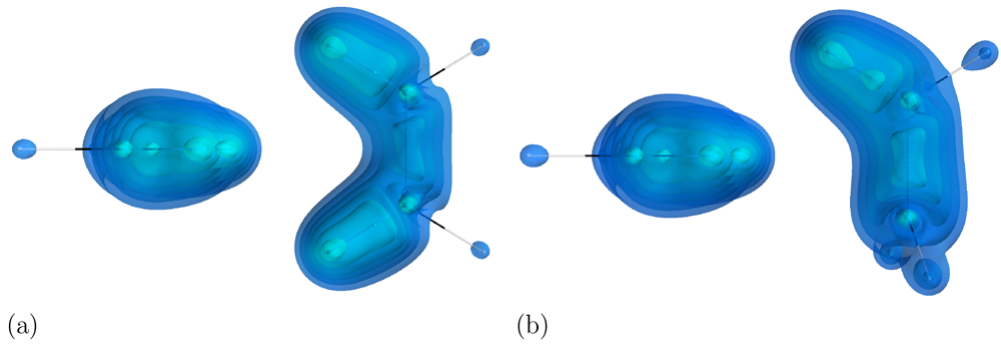
Visualization of the dispersion interaction density *D*^*AB*^ for ethylene dimer in the equilibrium geometry (a) and with one of the molecules with a CH_2_ group twisted by 90° (b). The six contours of isosurfaces of *D*^*AB*^(**r**) densities were generated to encompass 50%, 40%, 30%, 20%, 10%, and 1% of the integrated *D*^*AB*^(**r**), respectively.

To that end, let us look at the ethylene dimer in a staggered geometry (see Figure 2). It is well-established that this system is bound by the dispersion interaction with main contributions from the π–σ bond pairs and dihydrogen contacts.^43^ This interpretation is confirmed by the dispersion indicator (see Figure 2a). Clearly, the interaction originates from the electrons involved in the π bonds and C–H bonds that create the dihydrogen contacts, as illustrated by the absence of *D*^*AB*^ isosurface contours on the more distant C–H bonds in Figure 2a.

**Figure 3:**
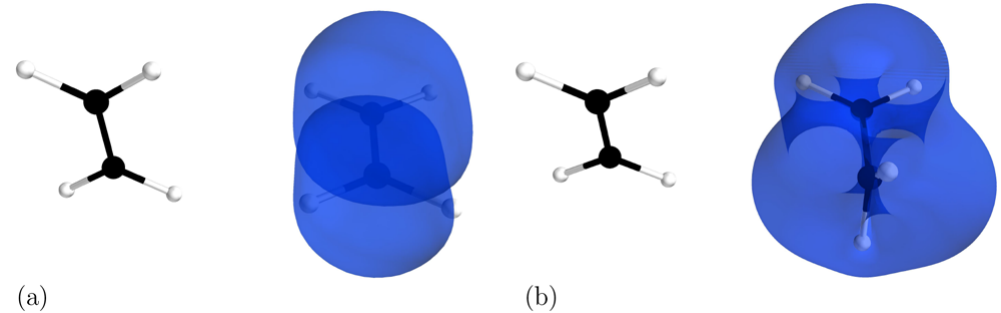
Visualization of (a) the geminal localized on the π bond in the ethylene molecule in its equilibrium geometry and (b) the corresponding geminal in the ethylene molecule with one CH_2_ group twisted by 90°. The contours encompass 90% of the geminal density.

When one twists the central π bond, one of the dihydrogen contacts disappears (see Figure 2b), and the π bond in the twisted molecule is destroyed (cf. Figure 3a,b). In the twisted ethylene, only the electrons comprising the C–H bonds and the ones formerly in the C–C bond are involved in the interaction. This destruction of a dihydrogen contact and the depletion of electrons from the C–C bond to other regions of the molecule leads to approximately a 40% decrease in binding according to EERPA-GVB and 20% according to SAPT(GVB). In the SAPT analysis, cf. Table 1, the magnitudes of three components of the interaction energy change significantly upon twisting: *E*_elst_^(1)^, that is, the electrostatic attraction diminishes; similarly, *E*_exch_^(1)^—the exchange repulsion and the attraction from the dispersion interaction *E*_disp_^(2)^ also lowers. However, the changes in the electrostatic and exchange terms cancel each other out so that the dispersion interaction decides about the change in the magnitude of the interaction.

**Table 1:** Interaction Energies of Ethylene (for Two Values of the Twisting Angle) and Diacetylene Dimers (for Two Intermonomer Distances) and Their Components (in kcal/mol)

	ethylene		diacetylene	
	0°	90°	4.26 Å	7.75 Å
GVB	0.54	0.72	0.75	–0.01
EERPA-GVB	–1.12	–0.64	–0.51	–0.04
SAPT(GVB)	–1.00	–0.81	–0.48	–0.04
*E*_elst_^(1)^	–0.64	–0.36	–0.33	–0.01
*E*_exch_^(1)^	1.28	1.00	1.19	0.00
*E*_ind_^(2)^	–0.16	–0.16	–0.44	0.00
*E*_exch–ind_^(2)^	0.12	0.13	0.36	0.00
*E*_disp_^(2)^	–1.74	–1.55	–1.40	–0.03
*E*_exch–disp_^(2)^	0.14	0.12	0.14	0.00
*E*_disp_^(2)^ + *E*_exch–disp_^(2)^	–1.60	–1.43	–1.26	–0.03
∑_*I*∈*A*_∑_*J*∈*B*_ε*_IJ_*	–1.80	–1.67	–1.36	–0.03

**Figure 4:**
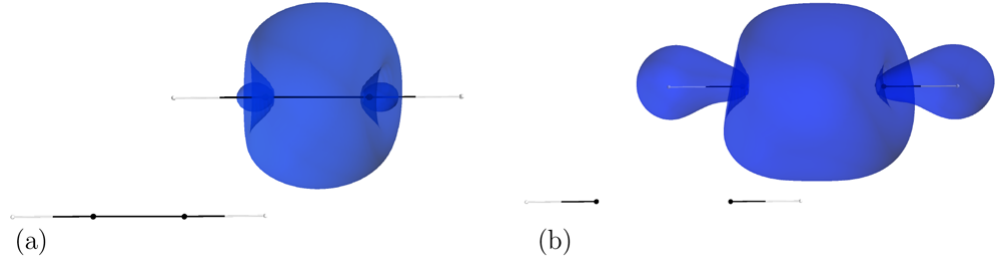
Visualization of the geminal localized on the C–C bond in the acetylene molecule in (a) its equilibrium geometry and (b) in stretched-bond geometry, at 2.04 Å C–C bond length. The contours encompass 90% of the geminal density.

**Figure 5:**
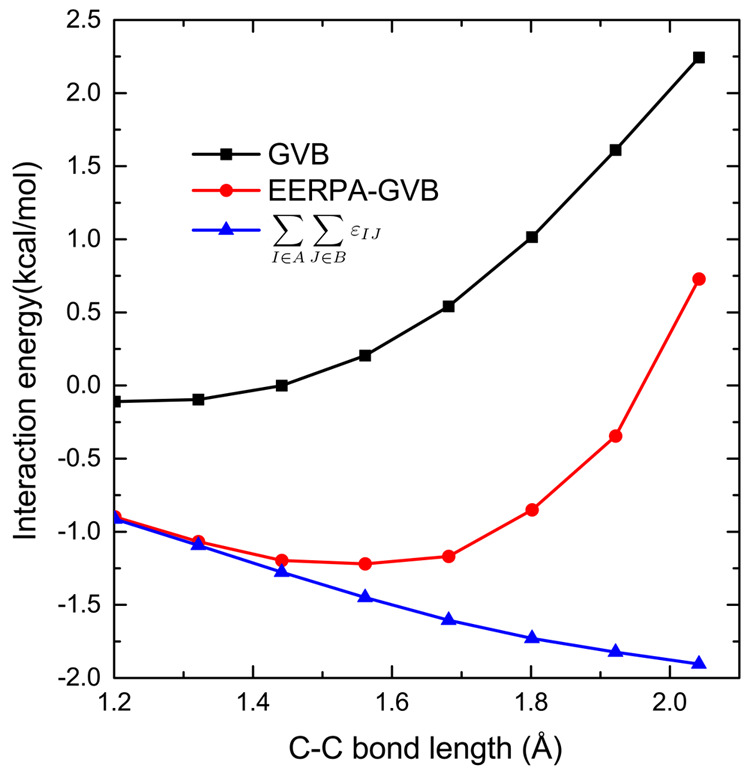
Interaction energy of the acetylene dimer for different C–C bond lengths.

Apart from twisting, a covalent bond can be also destroyed by stretching. As it is known, the GVB wave function is not capable of dissociating multiple bonds as a result of restricting orbitals in geminals to be singlet-coupled.^44^ It has been recently shown that GVB dissociation energy curves, corresponding to stretching multiple bonds, are improved upon adding an ERPA correlation energy correction.^45^ The EERPA-GVB method is therefore a viable tool to study a molecular interaction in such systems. An interesting example here is an acetylene dimer in a parallel-slipped geometry. The electrostatic and induction components are, to a large degree, compensated by the exchange and exchange-induction terms, and it is dispersion that plays a decisive role in binding at equilibrium geometry; see Table 2 and ref (46). With the C–C bonds stretched simultaneously in both monomers, the distribution of electrons comprising them becomes more diffuse, partly moving from the centers of molecules toward the C–H bonds (cf. Figure 4a,b). Since the π–π interaction is interrupted, one would expect that the stretching of C–C bonds leads immediately to a diminishing of the attraction, but actually, the opposite happens upon a small C–C bond elongation (see Table 2 and Figure 5). The interaction predicted by the GVB method, which roughly corresponds to the sum of the electrostatic, exchange, and induction components,^29^ is mildly attractive at the equilibrium geometry of ca. 1.2 Å and upon stretching becomes increasingly repulsive. This behavior of interaction energy is erroneous due to lack of the dispersion interaction in GVB. Looking at the SAPT(GVB) results, cf. Table 2, it can be seen that both the attractive and the repulsive components of the interaction grow (in absolute value), but while the growth of the exchange repulsion is much sharper than the one of the electrostatic attraction, the growth of dispersion is also very fast, which results in the overall increase of interaction when the C–C bonds are elongated. Only for very large bond lengths does the exchange again dominate, and, consequently, the intermonomer attraction decreases. In agreement with those results, in the EERPA-GVB analysis, the sum of the dispersion-like, cf. eq 16, negative in value terms grows monotonically. As a result of this interplay, the interaction energy has a minimum at ca. 1.5 Å.

**Table 2:** Interaction Energies and Their Components of Acetylene Dimer for Different C–C Bond Lengths (in kcal/mol) and Contributions to the Dispersion Energy from Fragments of the Monomers (in %)

*r*_CC_, Å	1.20	1.56	1.80	1.92
GVB	–0.33	0.20	1.01	1.61
EERPA-GVB	–1.16	–1.22	–0.85	–0.35
SAPT(GVB)	–1.07	–1.18	–0.79	–0.50
*E*_elst_^(1)^	–1.05	–2.50	–3.04	–3.22
*E*_exch_^(1)^	0.83	3.04	4.27	4.86
*E*_ind_^(2)^	–0.22	–1.07	–1.61	–1.87
*E*_exch–ind_^(2)^	0.16	0.90	1.41	1.66
*E*_disp_^(2)^	–0.87	–1.84	–2.22	–2.39
*E*_exch–disp_^(2)^	0.08	0.29	0.40	0.45
*E*_disp_^(2)^ + *E*_exch–disp_^(2)^	–0.79	–1.55	–1.82	–1.93
∑_*I*∈*A*_∑_*J*∈*B*_ε*_IJ_*	–0.91	–1.45	–1.73	–1.82
C–H (far)	2	2	1	1
C–H (close)	35	27	24	23
C–C	63	71	75	76

**Figure 6:**
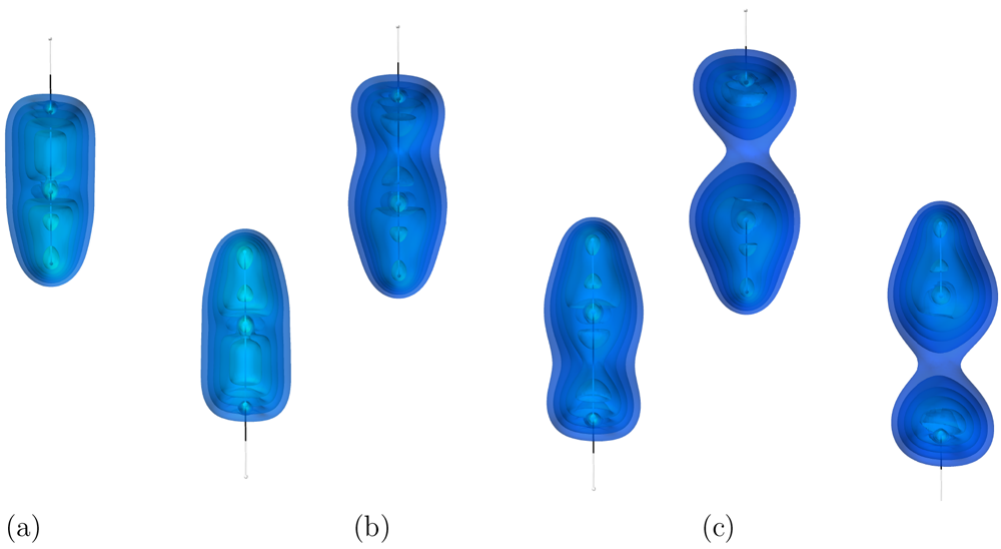
Visualization of the dispersion interaction density *D*^*AB*^ for the acetylene dimer in (a) its equilibrium geometry, (b) at 1.56 Å C–C bond length, (c) at 2.04 Å C–C bond length. The six contours of isosurfaces of *D*^*AB*^(**r**) densities were generated to encompass 50%, 40%, 30%, 20%, 10%, and 1% of the integrated *D*^*AB*^(**r**), respectively.

To understand this growth in the attraction, let us look at the dispersion interaction density in the dimer. In Figure 6, we see that, in the equilibrium geometry, most, ∼63% (see Table 2), of the dispersion interaction originates in the C–C bond areas, which is expected. The triple bonds are electron-rich, and in π bonds the electrons are relatively mobile, which results in high polarizabilities and a larger dispersion interaction.^47^ A significant fraction, ca. 35%, of the interaction comes also from the CH fragments closest to each other (see the entry “C–H (close)” in Table 2; the entry “C–H (far)” shows a contribution to dispersion energy from the outer CH fragments). In the stretched geometries, the π bonds become more strained and eventually are destroyed, and the electrons move closer to the carbon atoms. Consequently, most of the interaction comes from the electrons around the carbon atoms that are closest to each other. As the bond stretches further, for example, at 2.04 Å, the bond no longer exists, and each of the acetylene molecules becomes a pair of methylidyne radicals. The valence electrons, however, previously forming the CC bond, become more mobile and migrate outside of the bond region. Simultaneously, their polarizability grows even more, and therefore the dispersion interaction grows as well. As Table 2 shows, the electrons previously forming the bond continue to be the primary source of dispersion interaction, even when the bond no longer exists.

The growth of dispersion interaction upon the stretching of bonds in van der Waals (vdW) complexes and the appearance of a vdW minimum at stretched-bond geometries is consistent with our previous observations for similar systems^25,29^ and can impact the reactivity of those systems. For example, in an experiment using infrared radiation to excite a single vibrational mode,^48,49^ a growth or a decrease in the noncovalent interaction during such vibration can facilitate or block a reaction.

**Figure 7:**
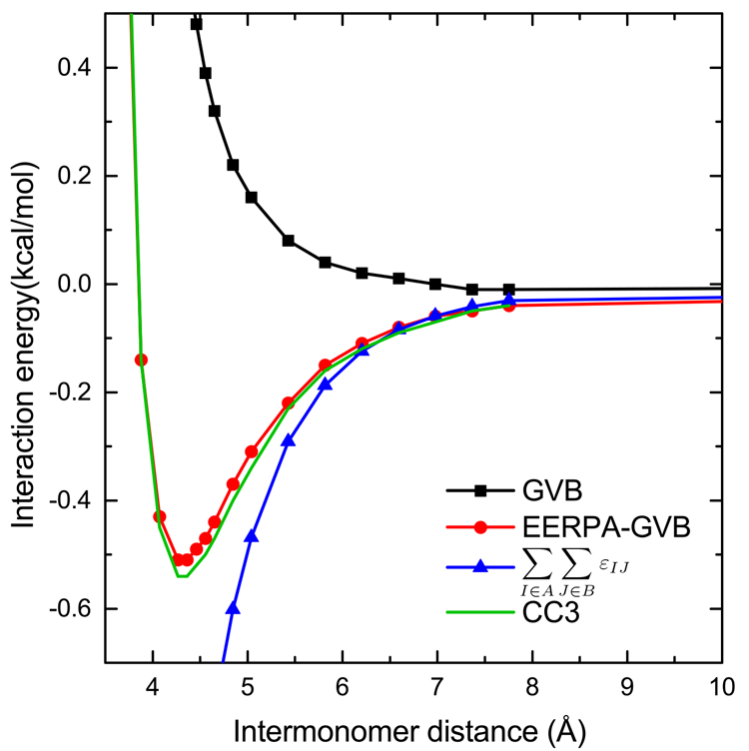
Interaction energy curve of the diacetylene dimer. The CC3 interaction energies are presented as a reference.

Finally, let us look at a diacetylene dimer. Diacetylene is the smallest representant of polyynes, that is, alkynes with alternating single and triple bonds. Polyynes are models of carbyne—the infinite carbon chain.^50,51^ In the interactions between polyynes, the key component of the interaction is believed to be the electrostatic attraction between the single and the triple bonds in the monomers.^33^ While this may be true for dimers comprising larger polyynes for diacetylene, where only a few such pairs are present, this is not the case. As is clear from Figure 7, the GVB approach, which already includes the electrostatic interaction, does not produce a binding curve. Only when the dispersion component is added, the interaction energy becomes negative; that is, an attraction appears. In agreement with this observation, the SAPT analysis in Table 1 shows that dispersion is the dominant attractive interaction here. In fact, the electrostatic component is very small, only −0.33 kcal/mol, and the much larger exchange repulsion is mainly counteracted by dispersion.

**Figure 8:**
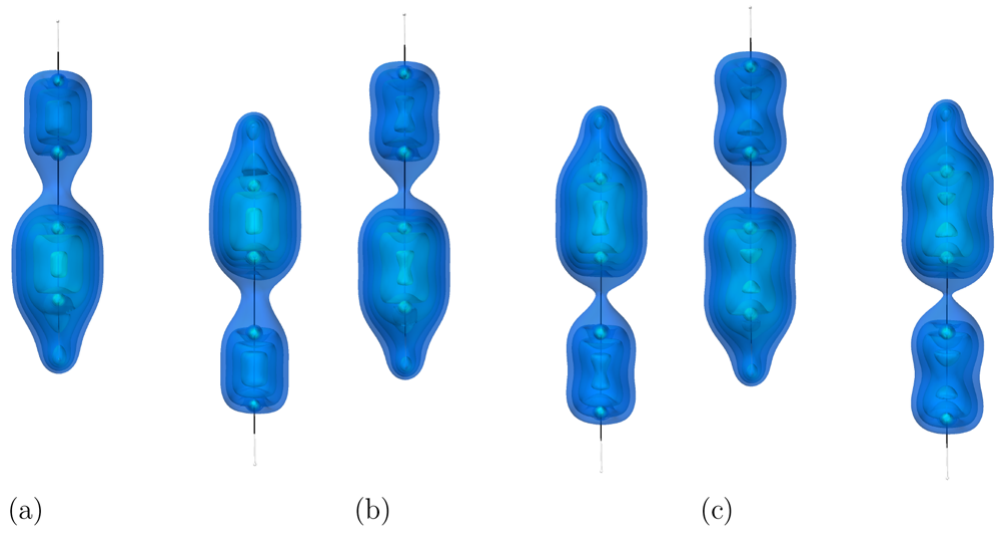
Visualization of the dispersion interaction density *D*^*AB*^ for the diacetylene dimer in (a) its equilibrium geometry, at 1.21 Å C–C triple bonds length, (b) at 1.33 Å C–C triple bonds length, (c) at 1.45 Å C–C triple bonds length. The six contours of isosurfaces of *D*^*AB*^(**r**) densities were generated to encompass 50%, 40%, 30%, 20%, 10%, and 1% of the integrated *D*^*AB*^(**r**), respectively.

The main source of the interaction between diacetylene molecules is the triple bonds in the molecules, as evidenced by a visualization of the *D*^*AB*^ descriptor, cf. Figure 8. This finding is not surprising, since the triple bonds are the most electron-rich regions of the monomers. As the bonds stretch, the interaction remains concentrated on the triple bonds closest to each other in the interacting monomers, and the *D*^*AB*^ local maxima move away from the single bond centers.

Thanks to the SAPT(GVB) analysis we can check if the dispersion  correlation term (i.e., the sum of the visualized contributions over the real space) matches the dispersion component as defined in SAPT. From Tables 1 and 2, we can see that this term is actually very close to the sum of the second-order dispersion and dispersion-exchange terms in SAPT(GVB). In agreement with eq 24, for large distances, when the overlap of electronic densities of the monomers is close to zero, the term  becomes equal to the true dispersion energy, while the exchange-dispersion term goes to zero (see the case of diacetylene dimer, for intermonomer distance 7.75 Å in Table 1).

## Conclusions

We introduced a new quantity designed to identify the regions of molecular systems that contribute the most to a dispersion-driven interaction. It is inspired by the Dispersion Interaction Density introduced by Wuttke and Mata,^17^ but instead of extracting the dispersion-like component from the local Møller–Plesset method, we use the analogous part of the correlation in the Embedded Extended Random Phase Approximation and exploit the localized nature of orbitals in the Perfect-Pairing Generalized Valence Bond method.

This change allows us to treat not only systems in their equilibrium geometries but also ones where bonds have been twisted, compressed, or stretched—even to the point of breaking. This opens the way to following the interactions between molecules undergoing chemical reactions as well as those in systems under pressure or strain.

As we showed in this work, the dispersion interaction visualization can be paired with the analysis of bonding patterns emerging from the geminal picture. The combination of those two methods can be therefore used to analyze both covalent and noncovalent interactions in a wide range of systems. The additional cost of this analysis is negligible, so it can be performed routinely along with EERPA-GVB computations. The analysis can be also paired with an SAPT(GVB) computation, which allows one to extract other components of the interaction, such as electrostatics, exchange, and induction.

To treat systems with significant multireference (other than one originating in the bond breaking) character, as well as excited states and open-shell systems, an extension of the description to AC-CASSCF, ERPA-CASSCF, or AC0-CASSCF^28,52,53^ methods is possible.

The analysis performed here for model systems confirms our observations^25,30^ that often during bond stretching the dispersion interaction in the complex can grow significantly, creating a van der Waals minimum with monomers out of their equilibrium geometries. We hope this conclusion can be used in designing experiments where the reaction is blocked or facilitated by exciting single vibration modes of substrate molecules.

## Supporting Information Available

Dalton.mol input files used in the computations for the article containing the Cartesian coordinates of moolecules in Figures 2–4, 6, and 8, and the basis sets used (ZIP)
